# Identifying training needs of practising community pharmacists in Jordan—a self-assessment study

**DOI:** 10.1186/s12913-024-11069-x

**Published:** 2024-06-10

**Authors:** Saja A. Alnahar, Rula M. Darwish, Shatha Z. Al Qasas, Mayada M. Al Shabani, Ian Bates

**Affiliations:** 1 Institute of Public Health, The University of Jordan, Amman, Jordan; 2 Honorary Research Fellow, Department of Primary Care and Public Health-Faculty of Medicine, Imperial College London, London, UK; 3 Department of Pharmaceutics and Pharmaceutical Technology, School of Pharmacy, University of Jordan, Amman, Jordan; 4 Training Department, The Jordanian Pharmacists Association, Amman, Jordan; 5 The Jordanian Pharmacists Association, Amman, Jordan; 6 Al Shaima’a Pharmacy, Amman, Jordan; 7 School of Pharmacy, University College London, London, UK

**Keywords:** Community pharmacists, Training priorities, CPD, Jordan, Clinical skills, Administrative skills, Interpersonal skills

## Abstract

**Background:**

Being the professional membership body for pharmacists in Jordan, the Jordan Pharmacists Association (JPA) took the initiative to establish a training centre for practising pharmacists. This study aims to identify the self-assessed training priorities of community pharmacists in Jordan.

**Methods:**

In the period between August and October 2022, an online self-administered questionnaire was distributed using a variety of participants’ identification and recruitment approaches. The questionnaire targeted currently practising community pharmacists. Data were analysed descriptively and inferentially.

**Results:**

In total, 470 community pharmacists participated in this study. Of 470 participants, 307 (65.3%) were employees, of which 206 were full-time employees. Results showed that only 97 (21%) had access to an in-house training programme or scheme. Self-assessment of training needs highlighted differences between the three competencies clusters. While administrative and managerial skills and competencies were more frequently prioritised on average than the other two clusters, interpersonal and communication skills were needed the least. Evidence showed a significant difference between female and male participants regarding the need for training addressing maternity and early childhood health training issues. Lastly, the role-based comparison showed that, compared to pharmacy owners, employees had a significantly higher need for training related to bookkeeping and taxation returns preparation and how to handle and manage records of narcotic and controlled medicines.

**Conclusions:**

If training and development programmes are tailored to address specific needs in administrative, clinical, and interpersonal competencies, community pharmacists have the potential to enhance public health, expand their role, provide patient-centred care, and support the national healthcare system.

**Supplementary Information:**

The online version contains supplementary material available at 10.1186/s12913-024-11069-x.

## Background

To combat poverty, preserve the environment, and improve human prospects [1], the United Nations (UN) announced its 2015 Sustainable Development Goals (SDGs), which address issues and challenges related to health, education, water sanitisation and nutrition [1]. The third goal aims to promote healthy living, achieve universal health coverage (UHC) and achieve equity in healthcare provision and access worldwide [1]. The World Health Organisation (WHO) based its Astana Declaration on three pillars of primary health care to support the implementation and achievement of the UN SDGs: (i) satisfying the health needs of a population by offering a wide range of promotive, protective, preventive, curative, rehabilitative, and palliative healthcare services throughout the life course; (ii) systematically addressing the broader determinants of health, including social, economic, and environmental contexts through evidence-based public policies and multi-sectoral action; and (iii) empowering individuals, families, and communities to optimise their health and supporting people like self-carers and caregivers as co-developers of health and social service [2, 3].

Achieving UN SDGs and implementing the WHO’s declaration requires competent and skilful healthcare professionals, high-quality and accessible healthcare services, advanced technologies, evidence-based practice and context-specific guidelines [4]. Among healthcare professionals and providers who can actualise the UN and WHO’s vision and agenda are pharmacists, especially those working in community and clinical settings.

Community pharmacists are uniquely positioned to assist and counsel the general public compared to other healthcare professionals. The vast majority of patients have ready and easy access to a pharmacy where they can get on-demand medical advice due to its convenient location and accessibility. Patients typically have high public trust and confidence in pharmacists’ advice regarding the prescription drugs that community pharmacists dispense and provide. Furthermore, pharmacists’ roles have grown to encompass more clinically focused duties [5]. Community pharmacists are, therefore, expected to possess the necessary competence, skill, and training to meet and satisfy the demands and needs related to public health and fulfil the expansion of their role. As a result, similar to other healthcare professionals, pharmacists should actively participate in continuing professional education (CPE) and continued professional development (CPD) [6].

In 2018, the Jordanian Government issued regulation No. 46, which discusses healthcare professionals’ license renewal requirements. As per the legislative piece and the subsequently released instructions, pharmacists, regardless of their sector of practice, are required to complete fifty credit hours over five years of continuous professional development (CPD) [7]. These credit hours may have different forms, including but not limited to attending training sessions, attending conferences and workshops, and participating in research projects [8].

Being the professional membership body for pharmacists in Jordan, the Jordan Pharmacists Association (JPA) took the initiative to establish a training centre for practising pharmacists. Furthermore, to have need-based training programmes, the JPA has asked the research team to identify training and development needs for practising community pharmacists in Jordan. The training needs of practising Jordanian community pharmacists have not been assessed before. In this study, the research team aims to assess and identify the training needs and priorities of Jordanian community pharmacists. Additionally, this study investigates training history of the targeted population and the availability of in-house training programmes or schemes.

This research can be used to create an action plan and set of recommendations to develop training materials and workshops for pharmacists to develop their skills and competencies.

## Methods

### Study design

This is a cross-sectional study targeting community pharmacists in Jordan. Eligible participants were currently practising community pharmacists who were identified and approached using the JPA database, social media platforms and relevant committees. Moreover, the research team relied on their personal connections to approach and recruit eligible participants.

### Study settings and participants

For this study, any practising community pharmacist in Jordan is considered eligible to participate. As per JPA records, there are 7,525 registered community pharmacists in Jordan. Accordingly, a sample size of 366 was considered to give adequate power for bivariate analysis to be carried out. The sample size was calculated based on a 50% expected frequency and a 5% confidence limit. Sample size was calculated using the OpenEpi platform. Moreover, as the primary data analysis approach is descriptive analysis, a sample size of 366 ensures the study results represent the population of community pharmacists in Jordan.

### Questionnaire design

Data were collected using a questionnaire instrument that developed by the research team. The instrument’s development was guided by the available relevant literature [9–20], study’s aim and objectives, and the feedback received from the JPA Training and Development Department and the JPA Continuous Professional Development Committee. Face and content validity of the instrument’s first version were assessed by academics and researchers specialised in pharmacy practice, senior community pharmacists and JPA training coordinator. Additionally, experts in training and human resources development were consulted. The questionnaire first version was amended as advised and recommended. Following experts review, the questionnaire’s comprehensiveness, readability and flow were assessed in a pilot study on a sample of thirteen pharmacists. Pilot study’s results were excluded from the final analysis.

The final questionnaire instruments consisted of fifteen questions groups into five groups as follows: first group was related to participant’s demographics and employment status. Second group was related to training history. Third group investigated training needs related to pharmaceutical care and clinical pharmacy practice skills and competencies. Fourth group was related to interpersonal and communication skills, and the fifth group was related to administrative and managerial skills.

### Survey distribution and data collection

In the period between August and October 2022, the validated and piloted questionnaire instrument was distributed and published using the Qualtrics XM® platform (Qualtrics, 2020). Practising community pharmacists in Jordan were sent the survey link via email, WhatsApp messages, and text messages.

A variety of participants’ identification and recruitment approaches were followed; these approaches were: (i) directly contacting eligible participants by the research team, (ii) posting an invitation to participate on the social media platforms of the JPA and its related committees, and (iii) contacting community pharmacists through the chairs of JPA regional committees on behalf of the research team.

### Statistical analysis

Collected data were extracted and logged in an Excel® workbook (Microsoft Office MS, 2013), which was used for data cleaning, coding and grouping.

Participants’ self-assessment of training needs were reported using a five-point Likert scale. Subsequent data aggregation was facilitated by scale conversion into a three-point scale. Accordingly, the first two categories (very high need and high need) were grouped into one (High Training Priority), the last two categories (very low need and low need) were grouped into one (Low Training Priority), the intermediate scale (Intermediate Priority) was left as it was.

Descriptive analysis in form of frequencies, percentages, and standard deviation was performed. Additionally, Z-test was performed to explore significant differences between different participants’ categories. Only completed surveys were considered for analysis and reporting.

### Ethical consideration

This study was reviewed and approved by the Institutional Review Board (IRB) of Yarmouk University (Reference No.: RD/119/12/3740). Participants were asked to consent to their participation electronically after being explained the study’s aim and objectives and their role and rights as participants. Only consenting participants took part in this study.

## Results

### Participants demographics and employment details

During three months of data collection, 470 community pharmacists completed the surveys. As electronic distribution and social media posting were the main distribution approaches, it was not possible to calculate response rate.

A total of 306 (65.1%) were females, and 77% were 45-year old or younger. The majority of the research participants were located in the Middle region. Out of 470 participants, 307 (65.3%) were employees, out of which 198 (64.5%) were employed by independent community pharmacies. Lastly, more than 55% of the participants had ten or less years of experience. Table 1 summaries participants’ demographics and employment details.

**Table 1:** Participants’ demographics, characteristics, employment details and training history

Investigated Attributes	Participants’ group		
	**Female participants** **N (%)**	**Male participants** **N (%)**	**Overall study participants** **N (%)**
**Demographics and characteristics**			
**Number of participants**	306 (65.1%)	164 (34.9%)	470 (100%)
**Participants’ age group**			
* 21 to 25*	60 (19.6%)	18 (11.0%)	78 (16.6%)
* 26 to 30*	81 (26.5%)	27 (16.5%)	108 (23.0%)
* 31 to 35*	37 (12.1%)	15 (9.1%)	52 (11.1%)
* 36 to 40*	33 (10.8%)	23 (14.0%)	56 (11.9%)
* 41 to 45*	41 (13.4%)	28 (17.1%)	69 (14.7%)
* 46 to 50*	30 (9.8%)	19 (11.6%)	49 (10.4%)
* 51 to 55*	15 (4.9%)	24 (14.6%)	39 (8.3%)
* 56 to 60*	4 (1.3%)	5 (3.0%)	9 (1.9%)
* 61 to 65*	5 (1.6%)	3 (1.8%)	8 (1.7%)
* Older than 65*	0 (0%)	2 (1.2%)	2 (0.4%)
**Region**			
* Northern*	68 (22.2%)	31 (18.9%)	99 (21.1%)
* Middle*	217 (70.9%)	125 (76.2%)	342 (72.8%)
* Southern*	21 (6.9%)	8 (4.9%)	29 (6.2%)
**Qualifications**			
** Degree**			
* BPharm*	284 (92.8%)	154 (93.9%)	438 (93.2%)
* PharmD*	22 (7.2%)	10 (6.1%)	32 (6.8%)
**Years of Experience**			
* Less than one year*	55 (18.0%)	8 (4.9%)	63 (13.4%)
* 1–5 years*	92 (30.1%)	31 (18.9%)	123 (26.2%)
* 6–10 years*	57 (18.6%)	21 (12.8%)	78 (16.6%)
* 11–15 years*	28 (9.2%)	30 (18.3%)	58 (12.3%)
* 16–20 years*	29 (9.5%)	26 (15.9%)	55 (11.7%)
* 21–25 years*	27 (8.8%)	19 (11.6%)	46 (9.8%)
* 26–30 years*	13 (4.2%)	12 (7.3%)	25 (5.3%)
* More than 30 years*	5 (1.6%)	17 (10.4%)	22 (4.7%)
**Employment details**			
** Employment status**			
* Full- time employee*	145 (47.4%)	61 (37.2%)	206 (43.8%)
* Part-time employee*	79 (25.8%)	22 (13.4%)	101 (21.5%)
* Pharmacy owner*	82 (26.8%)	81 (49.4%)	163 (34.7%)
**Type of community pharmacy**			
* Independent*	227 (74.2%)	128 (78.0%)	355 (75.5%)
* Chain*	79 (25.8%)	36 (22.0%)	115 (24.5%)
**Training history details**			
** Attended training activities in the past three years**			
* Yes*	203 (66.3%)	106 (64.6%)	309 (65.7%)
* No*	85 (27.8%)	55 (33.5%)	140 (29.8%)
* Not Sure*	18 (5.9%)	3 (1.8%)	21 (4.5%)
**Have a training record**			
* Yes*	97 (31.7%)	46 (28.0%)	143 (30.4%)
* No*	179 (58.5%)	104 (63.4%)	283 (60.2%)
* Not Sure*	30 (9.8%)	14 (8.5%)	44 (9.4%)
**Receive training-related notifications from the JPA**			
* Yes*	142 (46.4%)	72 (43.9%)	214 (45.5%)
* No*	128 (41.8%)	62 (37.8%)	190 (40.4%)
* Not Sure*	36 (11.8%)	30 (18.3%)	66 (14.0%)
**Have identified their training needs**			
* Yes*	135 (44.1%)	43 (26.2%)	178 (37.9%)
* No*	135 (44.1%)	106 (64.6%)	241 (51.3%)
* Not Sure*	36 (11.8%)	15 (9.1%)	51 (10.9%)
**Have an in house training programme**			
* Yes*	58 (19.0%)	39 (23.8%)	97 (20.6%)
* No*	226 (73.9%)	119 (72.6%)	345 (73.4%)
* Not Sure*	22 (7.2%)	6 (3.7%)	28 (6.0%)

*BPharm* Bachelor of Pharmacy, *JPA* Jordanian Pharmacists Association, *N* Number, *PharmD* Doctor of Pharmacy

### Training history and experiences

Participants’ training history and experiences were assessed in terms of attending training-related activities in the last three years, having a training records, identifying training needs and having an access to in-house training and development programme. Out of 309 (65.1%) respondents, who participated in training activities, 214 (69.3%) did not have an access to in-house training programme. Additionally, more than 50% received a notification from the JPA regarding training sessions, lectures or workshops. Results showed that less than one third of the study’s participants hold training records. Moreover, emerging evidence of Z-test showed that pharmacists employed by chain pharmacies were significantly more likely to have an in-house training programme, (*p* < 0.00001). lastly, less than 40% of the study’s participants were able to identify their training needs and priorities, Table 1.

### Training priorities

**Fig. 1:**
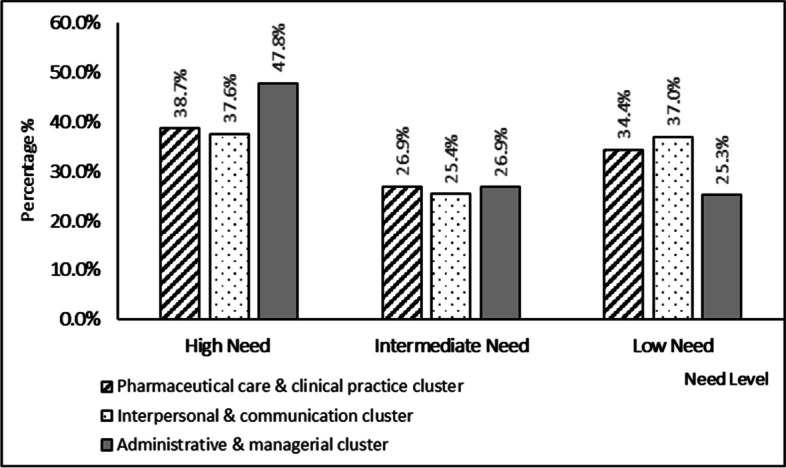
Comparison between the three competencies clusters

Self-assessment of training needs highlighted differences between the three competencies clusters. While, on average, administrative and managerial skills and competencies were more frequently prioritised over the other two clusters, interpersonal and communication skills were needed the least, Fig. 1.

#### Clinical pharmacy practice cluster

Within the clinical pharmacy practice cluster, 126 participants (26.8%) prioritised training in dispensing medicines and counselling special patient populations, such as the elderly, pregnant women, nursing mothers, and paediatric patients. Furthermore, more than 25% of participating pharmacists required training to read and interpret laboratory medical tests and dose adjustments accordingly. The findings indicated that about 30% of the participants expressed interest in attending training sessions on the proper and safe use of herbal remedies, dietary supplements, and vitamins. While Jordan is witnessing a surge in the prevalence of chronic and lifestyle diseases, only 96 (20%) of participating pharmacists reported a need for training related to chronic disease management and home-based diagnostic tests. Furthermore, 33% of the participants wanted additional training on extemporaneous preparations, compounding and selecting cosmetic products.

A closer look at the results shows that skills that require critical and analytical thinking, such as detecting and identifying drug-drug interactions and carrying out research and evaluating medicines-related information, research and literature, received the highest training priority within the clinical pharmacy cluster. Moreover, 170 (36.2%) participants reported a need for pharmacovigilance activities namely identifying and reporting medicines’ adverse drug reactions.

Lastly, a significant difference was observed between female and male participants regarding the need for maternity and early childhood health training, counselling and dispensing medicines to nursing and pregnant women, counselling regarding the selection of baby formulas and foods and dispensing and counselling regarding contraceptives.

#### Interpersonal and communication skills

While interpersonal and soft skills are considered integral for a successful professional life and career journey, they were prioritised the least by the research participants. Within this cluster, skills that might achieve harmony between team members, such as teamwork and conflict management, were more frequently reported as a training priority. Although, in theory, pharmacists working in chain pharmacies are expected to be more involved in team-based dynamics than those working in independent community pharmacies, evidence showed no significant differences in training priorities related to interpersonal skills and competencies. Differences between different groups were checked. Results showed that male pharmacists had a significantly higher priority of training related to effective communication. On the other hand, pharmacy owners had a significantly high priority of training addressing negotiation skills, Table 2.

**Table 2:** Training priorities within the three skills and competencies clusters

Skill/Competency	High priority	Average priority	Low priority
**Pharmaceutical care and clinical pharmacy practice skills**			
*Patient counselling*	175 (37.2%)	137 (29.1%)	158 (33.6%)
*Dispensing medicines and counselling elderly patients*	178 (37.9%)	135 (28.7%)	157 (33.4%)
*Dispensing medicines and counselling pregnant women*	197 (41.9%)	132 (28.1%)	141 (30.0%)
*Dispensing medicines and counselling nursing mothers*	200 (42.6%)	143 (30.4%)	127 (27.0%)
*Dispensing medicines and counselling fasting patients*	177 (37.7%)	126 (26.8%)	167 (35.5%)
*Dispensing medicines and counselling paediatric patients (younger than 16 years)*	168 (35.7%)	137(29.1%)	165 (35.1%)
*Dispensing medicines and counselling patients regarding contraceptives*	205 (43.6%)	134 (28.5%)	131 (27.9%)
*Dispensing medicines and counselling patients regarding cosmetic products and preparations*	223 (47.4%)	116 (24.7%)	131 (27.9%)
*Dispensing medicines and counselling patients regarding herbal remedies and preparations*	198 (42.1%)	148 (31.5%)	124 (26.4%)
*Dispensing medicines and counselling patients regarding narcotic medicines*	220 (46.8%)	109 (23.2%)	141 (30.0%)
*Dispensing medicines and counselling patients regarding smoking cessation products*	183 (38.9%)	144 (30.6%)	143 (30.4%)
*Dispensing medicines and counselling patients regarding vitamins and dietary supplements*	159 (33.8%)	132 (28.1%)	179 (38.1%)
*Dispensing medicines and counselling patients regarding weight management products*	168 (35.7%)	148 (31.5%)	154 (32.8%)
*Provide advice and counselling regarding the selection and use of baby formulas and foods*	146 (31.1%)	143 (30.4%)	181 (38.5%)
*Dispensing medicines and counselling patients regarding pain management medicines*	140 (29.8%)	134 (28.5%)	196 (41.7%)
*Management and treating minor ailments*	135 (28.7%)	110 (23.4%)	225 (47.9%)
*Interchangeability of medicines (generic-originator)*	118 (25.1%)	102 (21.7%)	250 (53.2%)
*Dispensing medicines and counselling patients regarding chronic diseases*	160 (34.0%)	118 (25.1%)	192 (40.9%)
*Training and guiding patients on how to use home-based diagnostic tests*	115 (24.5%)	84 (17.9%)	271 (57.7%)
*Reading and interpreting laboratory tests*	161 (34.3%)	115 (24.5%)	194 (41.3%)
*Vaccines administration*	175 (37.2%)	116 (24.7%)	179 (38.1%)
*Administrating medicines through the parenteral route (IM, SC, and IV)*	180 (38.3%)	100 (21.3%)	190 (40.4%)
*Extemporaneous preparation (compounding)*	219 (46.6%)	103 (21.9%)	148 (31.5%)
*Dosing calculation and adjustments*	182 (38.7%)	126 (26.8%)	162 (34.5%)
*Detecting and identifying drug-drug interactions*	240 (51.1%)	135 (28.7%)	95 (20.2%)
*Identifying medicines’ adverse drug reactions*	210 (44.7%)	146 (31.1%)	114 (24.3%)
*Reporting adverse drug reactions*	207 (44.0%)	143 (30.4%)	120 (25.5%)
*Carrying out research and evaluating medicines related information, research and literature*	231 (49.1%)	135 (28.7%)	104 (22.1%)
*Pharmacy practice and community pharmacy-related ethical and legal considerations*	173 (36.8%)	116 (24.7%)	181 (38.5%)
*Carrying out first aid procedures*	215 (45.7%)	128 (27.2%)	127 (27.0%)
**Interpersonal and communication skills**			
*Effective communication*	170 (36.2%)	124 (26.4%)	176 (37.4%)
*Negotiation skills*	196 (41.7%)	128 (27.2%)	146 (31.1%)
*Professional collaboration*	177 (37.7%)	126 (26.8%)	167 (35.5%)
*Teamwork*	149 (31.7%)	117 (24.9%)	204 (43.4%)
*Effective leadership*	191 (40.6%)	109 (23.2%)	170 (36.2%)
*Problem-solving*	185 (39.4%)	118 (25.1%)	167 (35.5%)
*Conflict management*	169 (36.0%)	115 (24.5%)	186 (39.6%)
**Administrative and managerial skills**			
*Digital literacy skills*	140 (29.8%)	146 (31.1%)	184 (39.1%)
*Bookkeeping and taxation returns preparation*	307 (65.3%)	88 (18.7%)	75 (16.0%)
*Financial management*	275 (58.5%)	124 (26.4%)	71 (15.1%)
*Insurance management*	242 (51.5%)	118 (25.1%)	110 (23.4%)
*Inventory management*	200 (42.6%)	123 (26.2%)	147 (31.3%)
*Sales and marketing*	216 (46.0%)	130 (27.7%)	124 (26.4%)
*Human resources*	194 (41.3%)	156 (33.2%)	120 (25.5%)
*Handling narcotic medicines records*	212 (45.1%)	104 (22.1%)	154 (32.8%)

#### Administrative and managerial skills

Regardless of its size and scope of activities, any organisation needs effective management to operate efficiently and meet its goals and objectives. The emerging evidence showed that skills and competencies within the administrative and managerial cluster were prioritised the most. Within this cluster, skills and know-how in bookkeeping, taxation returns preparation, and financial and insurance management were the most needed. Comparison between different groups revealed that independent pharmacy pharmacists had a significantly higher priority of training in insurance management. Moreover, the role-based comparison showed that employees had a significantly higher need for training related to bookkeeping and taxation returns preparation and how to handle and manage records of narcotic and controlled medicines, Table 2.

Finally, a comparison between young pharmacists, younger than 35 years old or less than five years of working experience, and senior pharmacists showed significant differences in training needs and priorities. While senior pharmacists prioritised digital literacy skills, all other skills within the administrative and managerial skills cluster were prioritised by young pharmacists.

## Discussion

As a profession, pharmacy is dynamic and requires continuous and responsive adaptation to the changing healthcare landscape nationally and internationally. Therefore, to ensure that current pharmaceutical practices and offered services can meet the general population’s needs, follow the most recent guidelines, and achieve efficiency in service delivery, there is a need to identify training and development priorities of practising community pharmacists. This study assessed the training needs and priorities of Jordanian community pharmacists. The study also investigated training history and community pharmacists’ access to an in-house training programme or system.

In total, 470 community pharmacists participated in the study using an electronic self-administered questionnaire. The questionnaire captured data on participants’ demographics, employment details, training history and the need for development and training related to pharmacy practice skills and competencies, interpersonal and communication skills, and administrative and managerial skills.

The survey started by assessing training history and training development-related arrangements and infrastructure. Starting in 2018, Jordanian healthcare professionals, including pharmacists, must complete 50 credit hours of continuous professional development (CPD) and submit a record of CPD activities. As a result, many professional and third-party organisations have actively developed, delivered and offered training courses and programmes. In this study, the impact of these initiatives was evident, as more than 65% of the participants attended training-related activities in the last three years. However, the current study showed that only one-third kept a record of their training activities. Interestingly and contradicting the findings of previous studies [9, 21], senior pharmacists were significantly more likely to keep a record of their training activities. Results indicate that the JPA and other responsible bodies, such as the High Health Council, need to consider initiatives encouraging pharmacists to record their training activities and offer platforms for pharmacists and other healthcare professionals to document and record their training and development activities.

Among the frequently reported barriers to CPE, CPD and training activities are accessibility, availability and affordability [10, 22]. One approach to overcoming these barriers is to access in-house training and development programmes. Implementing these programmes usually requires a well-established hierarchy, a common feature of chain pharmacies and not within independent small community pharmacies [23]. The results aligned with this notion, as independent pharmacy pharmacists are perceived to need help accessing training programmes. Accordingly, these pharmacists have limited opportunities for development and role expansion.

Pharmacists, as the custodians of medicines safety, are required to lead a proactive role in detecting, monitoring, and reporting adverse reactions to medicines and drug-drug interactions. Moreover, according to Schafheutle et al.’s literature review, competent and well-trained pharmacists could impact patients’ safety and the possibility of medicines-related errors [24]. Results showed that Jordanian community pharmacists prioritised skills related to medicine safety as training and development priorities. This could partly be correlated to the ageing population, the surge in lifestyle diseases, the growing number of poly-pharmacy patients and the introduction of new innovative medicines, which escalate the need for interventions and programmes that monitor patients’ safety and assure their overall well-being [23, 25].

Prior to the 2019 pharmacy programmes competencies framework, pharmacy programmes were either directed toward clinical practice, such as in the doctor of pharmacy (PharmD) programme, or chemistry and industrial content, such as bachelor of pharmaceutical science. Therefore, it was not surprising that administrative and managerial skills were prioritised the most, as these were not being taught or address during undergraduate formal academic programmes. Moreover, as community pharmacies sector is a for-profit business model, pharmacists could benefit from developing their administrative and managerial skills to assure efficient allocation of resources, smooth operation and customer satisfaction [23].

Lastly, surveyed pharmacists generally perceive training related to interpersonal and communication “Personal” competencies as the least needed. This finding could be attributed to the notion that personal competencies and skills can be developed with practice and time [9]. However, personal and communication skills are integral to general clinical practice and pharmacy practice. Pharmacists need to master these skills in order to communicate efficiently with their patients, colleagues and other healthcare professionals.

Results showed that there were gender-based differences that showed that the female participants gave more importance than the male participants to maternity and early childhood health training, counselling for nursing and pregnant women, and the dispensing and counselling of contraceptives. This highlights female pharmacists’ distinct roles and responsibilities and the gender-specific aspects of training needs. The difference could also be attributed to social and traditional considerations, as in a conservative culture, female patients might be more willing to approach female pharmacists for advice related to women’s health than male pharmacists. On the other hand, role-based differences showed that while pharmacy owners prioritsed negotiation skills. Lastly, compared to female pharmacists, male pharmacists valued effective communication. These results highlight the variety of training requirements depending on roles in the pharmacy industry.

As this is a self-administered questionnaire, there are several inherited design-related limitations. These limitations include the absence of open-ended questions, where participants share perceptions toward training activities or add skills and competencies other than the ones listed in the questionnaire. Additionally, the sample size and sampling approach could limit the generalisability of the study’s results and findings.

While this study aimed to assess the training priorities of community pharmacists, there is still a need for an accurate assessment of community pharmacists’ competency level. Although this study captured input from community pharmacists working in all governorates in Jordan, the majority of the study participants were located in Amman. This did not allow for a meaningful comparison between governorates or urban and rural locations.

Further research is needed to assess pharmacists’ competencies and skills objectively. Moreover, future research should investigate pharmacists’ perceptions toward CPD and CPE activities and preferred training approaches and channels. Lastly, an in-depth study might be needed to investigate the influence of gender and socioeconomic-related factors on training priorities and preferences.

## Conclusion

This study thoroughly assessed Jordanian community pharmacists’ top training priorities. The necessity for focused interventions in the training landscape is highlighted by the gaps in record-keeping, self-assessment, and access to internal training programmes that have been found. Community pharmacists could expand their role, deliver patient-centred care, support the national healthcare system and efficiently contribute to augmenting public health if training and development programmes are designed to meet specific needs in administrative, clinical, and interpersonal competencies. The gender and role-based disparities highlight the significance of customised training methods even more in order to guarantee a competent and well-rounded workforce in Jordan’s community pharmacy industry.

### Lessons for practice

- Community pharmacists need to assess their training and development needs and priorities periodically.
- Training priorities vary by gender, years of experience, employment status and type of community pharmacy.
- The insights gained from this study could be considered in developing and delivering national training programmes for community pharmacists in Jordan by the Jordanian Pharmacists Association or the High Health Council.

### Supplementary Information

Supplementary Material 1.

## Data Availability

The data that support the findings of this study are available on request from the corresponding author, Dr Saja A. Alnahar.

## Abbreviations

BPharm: Bachelor of Pharmacy
CPD: Continued Professional Development
CPE: Continuing Professional Education
IRB: Institutional Review Board
JPA: Jordanian Pharmacists Association
N: Number
PharmD: Doctor of Pharmacy Degree
SDGs: Sustainable Development Goals
UN: United Nations
WHO: World Health Organisation
