# Supplementary material for: Identifying training needs of practising community pharmacists in Jordan—a self-assessment study
Source: BMC Health Serv Res. 2024 Jun 10;24:712. doi: 10.1186/s12913-024-11069-x (PMC11163785; doi:10.1186/s12913-024-11069-x)
Supplement: Supplementary file 1 — Supplementary Material 1. [file 12913_2024_11069_MOESM1_ESM.docx]

# Title

Identifying Training Needs of Practicing Community Pharmacists in Jordan-A Self-Assessment Study-Questionnaire Template

**Corresponding Author:** Saja A. Alnahar-Institute of Public Health, The University of Jordan, Amman, Jordan, [s.alnahar@ju.edu.jo](mailto:s.alnahar@ju.edu.jo)

## Disclaimer

This questionnaire was developed, validated, piloted, and distributed in Arabic. Research questions, input from the professional leaders, feedback from the JPA training department, and the available literature guided the development of the questionnaire.

# Questionnaire Template

## Demographics and Characteristics

1. **Participant gender**
   1. Female
   2. Male
2. **Participant’s age group**
   1. 21 – 25 years old
   2. 26 – 30 years old
   3. 31 – 35 years old
   4. 36 – 40 years old
   5. 41 – 45 years old
   6. 46 – 50 years old
   7. 51 – 55 years old
   8. 56 – 60 years old
   9. 61 – 65 years old
   10. Older than 65 years
3. **Qualification degree**
   1. BPharm
   2. PharmD
4. **Governorate of residence**
   1. Amman (the capital)
   2. Irbid,
   3. Zarqa
   4. Mafraq
   5. Ajloun
   6. Jerash
   7. Madaba
   8. Balqa
   9. Karak
   10. Tafileh
   11. Maan
   12. Aqaba

## Employment Details

1. **Years of professional experience**
   1. Less than one year
   2. 1-5 years
   3. 6-10 years
   4. 11-15 years
   5. 16-20 years
   6. 21-25 years
   7. 26-30 years
   8. More than 30 years
2. **Employment status**
   1. Full-time employee
   2. Part-time employee
   3. Pharmacy owner
3. **Type of community pharmacy**
   1. Independent community pharmacy
   2. Chain community pharmacy

## Training history

1. **In the last three years, have you attending any training activities**
   1. Yes
   2. No
   3. Not sure
2. **Do you have a personal training record?**
   1. Yes
   2. No
   3. Not sure
3. **Have you ever received a training related notification from the Jordanian Pharmacists Association?**
   1. Yes
   2. No
   3. Not sure
4. **Have you ever identified your training needs?**
   1. Yes
   2. No
   3. Not sure
5. **Do you have an in house training programme at your pharmacy?**
   1. Yes
   2. No
   3. Not sure

## Training and development needs and priorities

### Pharmaceutical care and clinical pharmacy practice skills

1. **Please determine your training and development needs for the following tasks and knowledge.**

| **Skill/Competency** | Very High | High | Average | Low | Very low |
| --- | --- | --- | --- | --- | --- |
| Patient counselling |  |  |  |  |  |
| Dispensing medicines and counselling elderly patients |  |  |  |  |  |
| Dispensing medicines and counselling pregnant women |  |  |  |  |  |
| Dispensing medicines and counselling nursing mothers |  |  |  |  |  |
| Dispensing medicines and counselling fasting patients |  |  |  |  |  |
| Dispensing medicines and counselling paediatric patients (younger than 16 years) |  |  |  |  |  |
| Dispensing medicines and counselling patients regarding contraceptives |  |  |  |  |  |
| Dispensing medicines and counselling patients regarding cosmetic products and preparations |  |  |  |  |  |
| Dispensing medicines and counselling patients regarding herbal remedies and preparations |  |  |  |  |  |
| Dispensing medicines and counselling patients regarding narcotic medicines |  |  |  |  |  |
| Dispensing medicines and counselling patients regarding smoking cessation products |  |  |  |  |  |
| Dispensing medicines and counselling patients regarding vitamins and dietary supplements |  |  |  |  |  |
| Dispensing medicines and counselling patients regarding weight management products |  |  |  |  |  |
| Provide advice and counselling regarding the selection and use of baby formulas and foods |  |  |  |  |  |
| Dispensing medicines and counselling patients regarding pain management medicines |  |  |  |  |  |
| Management and treating minor ailments |  |  |  |  |  |
| Interchangeability of medicines (generic-originator) |  |  |  |  |  |
| Dispensing medicines and counselling patients regarding chronic diseases |  |  |  |  |  |
| Training and guiding patients on how to use home-based diagnostic tests |  |  |  |  |  |
| Reading and interpreting laboratory tests |  |  |  |  |  |
| Vaccines administration |  |  |  |  |  |
| Administrating medicines through the parenteral route (IM, SC, and IV) |  |  |  |  |  |
| Extemporaneous preparation (compounding) |  |  |  |  |  |
| Dosing calculation and adjustments |  |  |  |  |  |
| Detecting and identifying drug-drug interactions |  |  |  |  |  |
| Identifying medicines' adverse drug reactions |  |  |  |  |  |
| Reporting adverse drug reactions |  |  |  |  |  |
| Carrying out research and evaluating medicines related information, research and literature |  |  |  |  |  |
| Pharmacy practice and community pharmacy-related ethical and legal considerations |  |  |  |  |  |
| Carrying out first aid procedures |  |  |  |  |  |

### Interpersonal and communication skills

1. **Please determine your training and development needs for the following tasks and knowledge.**

| **Skill/Competency** | **Very High** | **High** | **Average** | **Low** | **Very low** |
| --- | --- | --- | --- | --- | --- |
| Effective communication |  |  |  |  |  |
| Negotiation skills |  |  |  |  |  |
| Professional collaboration |  |  |  |  |  |
| Teamwork |  |  |  |  |  |
| Effective leadership |  |  |  |  |  |
| Problem-solving |  |  |  |  |  |
| Conflict management |  |  |  |  |  |

### Administrative and managerial skills

1. **Please determine your training and development needs for the following tasks and knowledge.**

| **Skill/Competency** | **Very High** | **High** | **Average** | **Low** | **Very low** |
| --- | --- | --- | --- | --- | --- |
| *Digital literacy skills* |  |  |  |  |  |
| *Bookkeeping and taxation returns preparation* |  |  |  |  |  |
| *Financial management* |  |  |  |  |  |
| *Insurance management* |  |  |  |  |  |
| *Inventory management* |  |  |  |  |  |
| *Sales and marketing* |  |  |  |  |  |
| *Human resources* |  |  |  |  |  |
| *Handling narcotic medicines records* |  |  |  |  |  |
